# Ameliorating effect of 6-week swimming exercise on mice with experimental autoimmune encephalomyelitis (EAE) by reducing fetuin-A and increasing AMPK & NAD^⁺^ levels in liver tissue

**DOI:** 10.22038/IJBMS.2022.65117.14335

**Published:** 2022-08

**Authors:** Maryam Nazari, Mohammad Reza Kordi, Vazgen Minasian, Amir Hossein Saffar Kohneh Quchan

**Affiliations:** 1 Department of Exercise Physiology, Faculty of Physical Education and Sport Sciences, University of Isfahan, Isfahan, Iran; 2 Department of Exercise Physiology, Faculty of Physical Education and Sport Sciences, University of Tehran, Tehran, Iran

**Keywords:** AMPK, Exercise, Experimental autoimmune – encephalomyelitis, Fetuin-A, NAD⁺

## Abstract

**Objective(s):**

Multiple sclerosis (MS) is a chronic inflammatory disease affecting sensory and motor function in the central nervous system. Physical activities in the prevention and treatment of such conditions have shown promising results. However, their mechanisms of action have not been fully known yet and need further study. The present study aimed to evaluate the preventive effect of swimming exercise on some liver factors involved in inflammation and MS.

**Materials and Methods:**

In this study, experimental autoimmune encephalomyelitis was induced in C57BL/6 mice, and the effect of a 6-week swimming exercise on the levels of fetuin-A, AMP-activated protein kinase (AMPK), and Nicotinamide adenine dinucleotide (NAD^+^) in their liver tissue was investigated by western blot analysis and NAD^+^ colorimetric assay.

**Results:**

The study showed that EAE induction substantially (3.5 - fold) enhanced the fetuin-A levels and caused a reduction in AMPK and NAD^+^ amount. This is when doing 6 weeks of swimming exercise reduced fetuin-A to slightly above control. Also, levels of AMPK and NAD^+^ markedly increased in C57BL/6 mice with EAE.

**Conclusion:**

Doing regular exercise may limit the body’s inflammatory responses and reduce the severity of MS by regulating the expression of fetuin-A and increasing AMPK and NAD^⁺^ levels in liver tissue.

## Introduction

Multiple scleros (MS) is a progressive and often debilitating disease of the nervous system. This disease causes degradation of the myelin sheath of nerve cells, disrupting the direction of the nervous messages in the central nervous system (1). The cause of MS is not fully known, but it seems that activation of autoimmune and inflammation play a central role in causing the disease (2). Identifying MS biomarkers can help the early diagnosis of the disease and minimize the occurrence of disability in the future and the progression of its complications.

Alpha-2-HS-glycoprotein, also known as fetuin-A, is a 64-kDa phosphorylated glycoprotein specifically secreted by the liver cells. It is one of the MS biomarkers that directly indicate the disease activity (3, 4). Recently, a study by Harris *et al.* has shown that cerebrospinal fluid fetuin-A levels significantly increased in patients with MS. The increased expression of fetuin-A was also observed in damaged and demyelinated areas of the spinal cord and brain tissue of rats with experimental autoimmune encephalomyelitis (EAE) (3). 

The precise mechanism by which fetuin-A may affect the pathogenesis of EAE or MS is not understood. However, recent studies have shown that fetuin-A plays a key role in attenuating two metabolic sensors, SIRT_1 _(member of the sirtuin family) and AMP-activated protein kinase (AMPK), a heterotrimeric complex involved in cellular energy sensing, regulating metabolism, and adapting exercise. An inverse linear relationship has been observed between Fetuin-A and AMPK levels (5), It has been indicated that fetuin-A as an upstream factor can degrade SIRT_1 _and reduce AMPK levels. *In vivo* studies also confirm that reduction of fetuin-A increases AMPK levels (6). Recently, the role of AMPK has been considered in aging and some diseases, such as MS and diabetes. A reduction in AMPK has been observed in all cells of the immune system of rats with EAE (7). These findings suggest that AMPK recovery can be a new therapeutic target in autoimmune diseases such as MS.

In addition to AMPK, nicotinamide adenine dinucleotide (NAD⁺) is a coenzyme involved in cellular metabolism and plays a vital role in mitochondrial function and calcium homeostasis (8). Most initial research has been conducted on the contribution of NAD⁺ to mitochondrial adaptation to exercise. In addition, therapeutic applications of NAD⁺ and its involvement in multiple signaling pathways have been considered recently. Investigations have demonstrated that the NAD⁺ treatment can inhibit inflammatory responses by activating the AMPK/SIRT_1_ pathway (9). Therefore, the effectiveness of NAD⁺ in the MS treatment process should not be overlooked.

Exercise as a non-pharmacological intervention plays a critical role in preventing and treating neurological diseases such as MS (10). Studies have shown that NAD⁺, fetuin-A, and AMPK levels change in response to exercise and nutrition. For example, Zhang *et al*. (2019) showed that after eight weeks of aerobic exercise on a treadmill, the expression of mRNA and phosphorylated AMPK (p-AMPK) in rat skeletal muscles increased significantly (11). Likewise, Costford *et al*. (2010) reported that three weeks of exercise could increase NAD⁺ in the muscles (12). Also, the effect of exercise on reducing fetuin-A in individuals with diabetes and obesity has been reported previously (13, 14). In a 2017 study, plasma fetuin-A levels increased after six months of exercise with weight loss in obese individuals (15), while Zhang *et al*. (2018) reported that 12 weeks of endurance exercise significantly reduced fetuin-A serum levels in diabetic patients (14). However, the effect of exercise on fetuin-A expression in MS has not been studied yet.

Regarding the importance of fetuin-A, AMPK, and NAD in MS and the potential effect of exercise on this disease, the present study aimed to investigate the impact of 6-week swimming exercise on the expression of the fetuin-A protein, AMPK, and NAD⁺ in the liver tissue of C57BL/6 mice with EAE.

## Materials and Methods

Twenty-four female 6–8 week old C57BL/6BL6 mice weighing 16–20 g were purchased from the Pasteur Institute of Iran. In order to adapt to the new condition, the animals were kept in the animal house for 2 weeks in 12:12 hr dark/light. Then, the animals were randomly divided into 3 groups (n = 8): healthy control (control), EAE induced (EAE), and EAE induced subjected to swimming exercise (EAE+E). All C57BL/6 mice were housed under standard conditions (21 ± 2 °C and 45–55% humidity) with free access to food and water. The Ethics Committee of Tehran Medical School approved all the testing procedures (IR.UT.SPORT.REC.1397.028)

***Exercise protocol***

**Figure 1:**
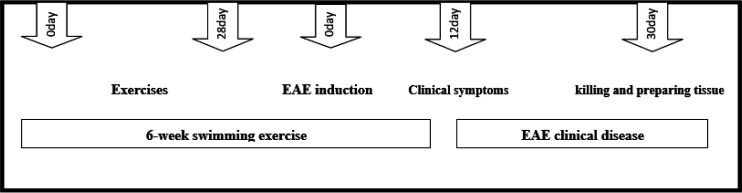
This figure illustrates different stages of exercise, EAE induction, symptom onset, and animal sacrifice. In this experiment, the impact of a 6-week swimming exercise on the expression of the fetuin-A protein, AMPK, and NAD⁺ in the liver tissue of C57BL/6 mice with EAE was evaluated

The animals of the EAE+E group performed the endurance swimming program for 6 weeks; 5 days per week, 30 min every day at 31 ± 1 °C. At the same time, the control group was placed in a water cage while their feet were on the bottom of the cage. The EAE induction process was started 48 hr after the last exercise session of the fourth week, and then, the exercises continued for 12 days until the appearance of clinical symptoms. The process of performing the stages is shown in Figure 1. 

***EAE induction***

The induction of the EAE model was successfully produced at the Salari Institute of Cognitive and Behavioral Disorders (SICBD). Toward this end, at first, mice were immunized by subcutaneous injection of 200 µg of myelin oligodendrocytes glycoprotein (MOG35-55) dissolved in phosphate-buffered saline (PBS) and emulsified with an equal volume of complete Freund’s adjuvant (CFA) supplemented with 400 µg of *Mycobacterium tuberculosis* H37Ra extract in small and stable particles. Next, 300 ng of pertussis toxin was injected intraperitoneally into C57BL/6 mice of EAE and EAE+E groups twice with an interval of two days. It should be noted that the control group received saline only.

***EAE clinical symptoms assessments***

Animals’ body weight was evaluated daily as a health parameter. The EAE clinical symptoms were also assessed by two independent observers and an EAE clinical scoring system was used to evaluate neurological disorders in the EAE model according to the following scale: Score 0 = no disease; Score 1 = weight loss and weakness in the tail; Score 2 = weakness in the hind limbs; Score 3 = complete hind limb paralysis; Score 4 = hind limb paralysis with weakness or paralysis in the front limb; and Score 5 = animal death.

**Molecular studies**

***Protein extraction***

The C57BL/6 mice were killed 30 days after EAE induction (in the chronic period of the disease), and their liver tissue was collected. Next, the lysis buffer was used to extract liver tissue proteins, and the samples were centrifuged at 4 °C and 12,000 rpm for 10 min. The supernatant containing proteins was extracted and stored at -20 °C.

***Western blot analysis***

The fetuin-A and AMPK levels in liver tissue were measured using the Western blot analysis. In order to perform this technique, equal amounts of the extracted protein were loaded on the SDS-PAGE polyacrylamide gel (12%). After electrophoresis, the protein bands separated on the gel were transferred to a PVDF membrane, followed by immersing the membrane in the blocking solution (nonfat dried milk diluted in TBST) for 75 min. The membrane was then exposed to the primary anti-AMPK and anti-fetuin-A antibodies (Santa Cruz Co, USA) for 24 hr at 4 °C. Subsequently, it was washed three times with TBST solution and treated for 75 min with secondary anti rabbit antibody at the concentration of 1: 1000. After washing with TBST, the blots were covered with ECL reagents and identified using LI-COR scanner (USA). Beta-actin was also considered as a loading control. 

***NAD*** ***⁺*** ***assay***

A NAD/NADH Quantification Kit (MAK037; Sigma-Aldrich, Germany) was used to measure NAD⁺ levels according to the manufacturer’s instructions.

***Statistical analysis***

Statistical analysis of data was performed by SPSS software version 21. One-way ANOVA was used to determine the differences between the means of the groups, and Tukey’s *post hoc* test was used to determine the differences between the groups. The clinical score and body weight were analyzed by Mann-Whitney test. *P*<0.05 was considered significant. 

EAE: encephalomyelitis

**Table 1:** Descriptive statistics of the bodyweight and clinical score on days 12 (onset sign of disease) and 30 (chronic period of the disease) post-immunization

Disease parameter	EAE	Swimming	*P*≤0.05
Clinical score at day 12	0.5 ± 0.53 (0-1)	0.33 ± 0.49 (0-1)	0.46
Clinical score at day 30	2.0 ± 1.0 (1-3)	1.5 ± 1.19 (0-3)	0.44
Weight at day 12	17 ± 1.0 (16-18)	18.16± 1.8 (16-22)	0.13
Weight at day 30	16.80 ± 1.09 (16-18)	18.25 ± 2.91 (14-22)	0.22

**Figure 2:**
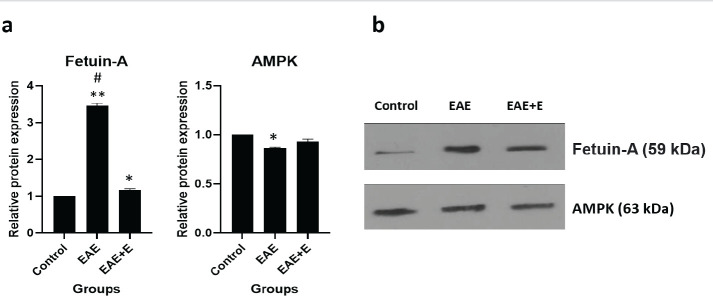
Western blot analysis results. The relative expression of fetuin-A, AMPK, and B-actin compared with the control group (a) and the bands related to each protein (b). One-way analysis of variance for fetuin-A expression confirmed the significant difference between the three groups. * and ** show significant differences between control and treatment groups with *P*˂0.05 and *P*˂0.001, respectively. # shows significant differences between EAE and EAE+E groups with *P*˂0.01

EAE: encephalomyelitis

**Figure 3:**
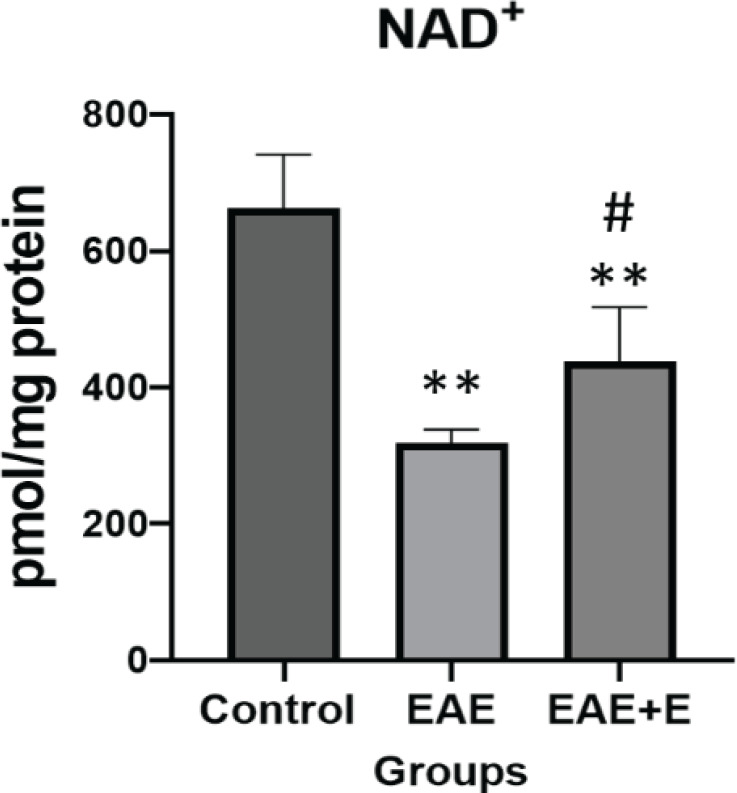
This figure shows the NAD⁺ amount in the control, EAE, and EAE+E groups. The statistical analysis of variance related to NAD^⁺ ^molecule showed a significant difference between the three groups. The results of Tukey *post hoc* test showed a significant difference between the control and EAE groups (**P=0.001), control and EAE +E groups (** *P*=0.001) and EAE and EAE + E groups (# *P*=0.039)

EAE: encephalomyelitis

## Results

***EAE clinical symptoms***

As shown in Table 1, on day 12 after EAE induction, the animals showed clinical signs of disease. Following swimming, a reduction was observed in the EAE + swimming group’s clinical scores compared with the EAE group at the onset (EAE: 0.5 ± 0.53, Swimming: 0.33 ± 0.49, *P*=0.46) and chronic period (EAE: 2.0 ± 1.0, Swimming: 1.5 ± 1.19, *P*=0.44) of the disease but this reduction was not significant. 

***NAD*** ***⁺*** ***assay***

The NAD⁺ assay results revealed that the NAD⁺ levels in the control group were about 650 pmol/mg protein, while the amount of this compound in the EAE group was half that amount. This feature showed a considerable increase in the EAE+E group and reached about 450 pmol/mg protein (Figure 3).

## Discussion

The present study aimed to evaluate the effect of exercise on the expression of fetuin-A, AMP-activated protein kinase, and Nicotinamide adenine dinucleotide (NAD⁺) in the liver tissue of mice with experimental autoimmune encephalomyelitis (EAE). The study results showed that EAE induction could increase the expression of fetuin-A while reducing the AMPK and NAD^+^ amount. On the other hand, swimming significantly reduced fetuin-A expression and increased the levels of AMPK and NAD^+^ in the animals with EAE.

Few studies have currently investigated the effect of exercise on liver fetuin-A levels. However, several studies have recently reported the positive impact of exercise on reducing serum levels of fetuin-A. A study by Sakr *et al*. showed that after 16 weeks of swimming, serum fetuin-A decreased significantly in rats with metabolic syndrome (16). Keihanian *et al.* have done a similar investigation on humans and reported similar results. Their study revealed a significant reduction in serum fetuin-A in diabetic patients after eight weeks of aerobic and resistance exercise (17).

In recent years, the liver has been considered an endocrine organ because it releases certain proteins, most of which play essential roles in metabolic homeostasis, inflammation, and diseases(18, 19). fetuin-A is a hepatokine secreted by the liver and known as an endogenous ligand for Toll-Like Receptor 4 (TLR4). This protein is encoded by the *ahsg* gene and expressed in many organs such as the liver, adipose tissue, and skeletal muscle (20). It is clear now that regular exercise benefits inflammatory diseases such as obesity, NAFLD, type 2 diabetes, and MS (18, 21). Therefore, it can be assumed that the reduction in fetuin-A through the TLR4 pathway, as a result of exercise, is a mechanism involved in the anti-inflammatory process.

On the other hand, our results showed a reduction in AMPK expression after EAE induction and a significant increase in AMPK protein expression in mice that exercised. The observed rise in AMPK is consistent with the results of previous studies (11, 22). Morissette *et al*. observed a significant increase in phosphorylated AMPK and total AMPK content in cardiac tissue after five months of rat exercise by merry-go-round (22). Zhang *et al*. also reported that after 8 weeks of treadmill aerobic exercise, the expression of AMPK and related mRNA increased significantly in rats (11). AMPK is an energy-sensitive enzyme in all cells of the mammalian body. During exercise, AMPK is activated in skeletal muscle, adipose tissue, liver, and other organs by increasing AMP/ATP ratio (23). Exercise is recognized as the most potent physiological activator of AMPK (24), and the change in expression of this protein is a unique model for studying many physiological roles of exercise. Exercise plays a mediating role in activating AMPK, which can prevent and treat some diseases such as MS.

One of the other observations of our study was a significant increase in NAD⁺ following swimming exercises. NAD⁺ is essential for the oxidative phosphorylation process in cells and acts as an electron shuttle between tricarboxylic acid (TCA) and electron transport chain (ETC) cycles (25). So, NAD⁺ is one of the key molecules involved in regulating the body’s metabolism and homeostasis. Endogenous NAD^+ ^and total levels of this enzymatic cofactor reduce in some chronic and degenerative diseases such as Alzheimer’s, Parkinson’s, muscular dystrophy, and cardiovascular diseases (26). Many studies have shown that the increase of NAD⁺, either biologically or through dietary supplements, is a promising strategy for achieving its multifaceted health advantages (27). NAD⁺ can support protective and compensatory processes in central nervous system diseases, such as MS (9). Wang *et al*. have recently reported that daily injection of NAD⁺ in rats with EAE ameliorated nerve damage and improved movement disorders. In addition, it has been demonstrated that NAD⁺ treatment reduced the expression of pro-inflammatory factors, such as IL-2, IL-17, and IL-18, and increased the expression of anti-inflammatory IL-10 (28). Therefore, it can be said that NAD⁺ relieves EAE symptoms by reducing inflammation and can be an effective treatment strategy for MS.

In general, nicotinamide phosphoribosyltransferase (NAMPT) is responsible for converting nicotinamide to NAD⁺ in mammals. And the increased NAMPT expression increases NAD⁺ molecule in cells (29). Studies have shown that active people have more NAMPT and NAD⁺ in their muscles than inactive people. After 3 weeks of exercise intervention, NAMPT and NAD⁺ molecule increases in inactive people too (12, 30). Activation of AMPK has also been shown to increase NAMPT mRNA. Therefore, the exercise by increasing the expression of AMPK and NAMPT can regulate downstream pathways, such as increasing NAD⁺ (12). There are few studies on the effect of exercise on NAD⁺ in different body tissues; however, our results suggest that doing exercise can increase liver NAD⁺ and may effectively reduce inflammation and limit the severity of EAE. 

## Conclusion

The present study revealed that 6-weeks of exercise could significantly regulate the increased levels of fetuin-A in C57BL/6 mice with EAE. It could also increase the AMPK and NAD⁺ levels. Considering these proteins play a central role in chronic inflammatory disease, such as MS, exercise in this way very likely induces its influence, preventing the incidence of these diseases and ameliorating their effects.

## Authors’ Contributions

MN and MRK Designed the experiments; MN and Ahskq Performed experiments and collected data; MN, MRK and VM Discussed the results and strategy; VM and MRK Supervised, directed and managed the study; MN, MRK, VM, and Ahskq Final approved of the version to be published.

## Ethical Approval

This study was carried out in accordance with the ethical code of working with laboratory animals, under IR.UT.SPORT.REC.1397.028 code of the University of Tehran.

## Conflicts of Interest

None.

## References

[1] Lubetzki C, Stankoff B (2014). Demyelination in multiple sclerosis. Handb Clin Neurol..

[2] Gandhi R, Laroni A, Weiner HL (2010). Role of the innate immune system in the pathogenesis of multiple sclerosis. J Neuroimmunol.

[3] Harris VK, Donelan N, Yan QJ, Clark K, Touray A, Rammal M (2013). Cerebrospinal fluid fetuin-A is a biomarker of active multiple sclerosis. Mult Scler.

[4] Aroner SA, Mukamal KJ, St-Jules DE, Budoff MJ, Katz R, Criqui MH (2017). Fetuin-A and risk of diabetes independent of liver fat content: the multi-ethnic study of atherosclerosis. Am J Epidemiol.

[5] Chattopadhyay M, Mukherjee S, Chatterjee SK, Chattopadhyay D, Das S, Majumdar SS (2018). Impairment of energy sensors, SIRT1 and AMPK, in lipid induced inflamed adipocyte is regulated by fetuin A. Cell Signal.

[6] Jung TW, Ahn SH, Shin JW, Kim HC, Park ES, Abd El‐Aty A (2019). Protectin DX ameliorates palmitate‐induced hepatic insulin resistance through AMPK/SIRT 1‐mediated modulation of fetuin‐A and SeP expression. Clin Exp Pharmacol Physiol.

[7] Nath N, Khan M, Rattan R, Mangalam A, Makkar RS, de Meester C (2009). Loss of AMPK exacerbates experimental autoimmune encephalomyelitis disease severity. Biochem Biophys Res Commun.

[8] Tullius SG, Biefer HRC, Li S, Trachtenberg AJ, Edtinger K, Quante M (2014). NAD+ protects against EAE by regulating CD4+ T-cell differentiation. Nat Commun.

[9] Wang J, Zhao C, Kong P, Sun H, Sun Z, Bian G (2016). Treatment with NAD+ inhibited experimental autoimmune encephalomyelitis by activating AMPK/SIRT1 signaling pathway and modulating Th1/Th17 immune responses in mice. Int Immunopharmacol.

[10] Close GL, Hamilton DL, Philp A, Burke LM, Morton JP (2016). New strategies in sport nutrition to increase exercise performance. Free Radic Biol Med.

[11] Zhang Y-J, Li J, Huang W, Mo G-Y, Wang L-H, Zhuo Y (2019). Effect of electroacupuncture combined with treadmill exercise on body weight and expression of PGC-1α, Irisin and AMPK in skeletal muscle of diet-induced obesity rats. Zhen Ci Yan Jiu.

[12] Costford SR, Bajpeyi S, Pasarica M, Albarado DC, Thomas SC, Xie H (2010). Skeletal muscle NAMPT is induced by exercise in humans. Am J Physiol Endocrinol Metab.

[13] Ren G, Bowers RL, Kim T, Araya‐Ramirez F, Mahurin AJ, Dean DM (2020). Alterations of serum ser312‐phosphorylated fetuin‐A from exercise‐induced moderate body weight loss in individuals with obesity. Obesity.

[14] Zhang L-Y, Liu T, Teng Y-Q, Yao X-Y, Zhao T-T, Lin L-Y (2018). Effect of a 12-week aerobic exercise training on serum fetuin-A and adipocytokine levels in type 2 diabetes. Exp Clin Endocrinol Diabetes.

[15] Blumenthal JB, Gitterman A, Ryan AS, Prior SJ (2017). Effects of exercise training and weight loss on plasma fetuin-a levels and insulin sensitivity in overweight older men. J Diabetes Res.

[16] Sakr HF, Al-Hashem FH, El-Naby WMH, Alkhateeb MA, Zaki MSA, Refaey HME (2014). Preventive roles of swimming exercise and pioglitazone treatment on hepatic dysfunction in a rat model of metabolic syndrome. Can J Physiol Pharmacol.

[17] Keihanian A, Arazi H, Kargarfard M (2019). Effects of aerobic versus resistance training on serum fetuin-A, fetuin-B, and fibroblast growth factor-21 levels in male diabetic patients. Physiol Int.

[18] Ennequin G, Sirvent P, Whitham M (2019). Role of exercise-induced hepatokines in metabolic disorders. Am J Physiol Endocrinol Metab.

[19] Hoene M, Franken H, Fritsche L, Lehmann R, Pohl A, Häring H (2010). Activation of the mitogen-activated protein kinase (MAPK) signalling pathway in the liver of mice is related to plasma glucose levels after acute exercise. Diabetologia.

[20] Pal D, Dasgupta S, Kundu R, Maitra S, Das G, Mukhopadhyay S (2012). Fetuin-A acts as an endogenous ligand of TLR4 to promote lipid-induced insulin resistance. Nat Med.

[21] Quchan AHSK, Kordi MR, Namdari H, Shabkhiz F (2020). Voluntary wheel running stimulates the expression of Nrf-2 and interleukin-10 but suppresses interleukin-17 in experimental autoimmune encephalomyelitis. Neurosci Lett.

[22] Morissette MP, Susser SE, Stammers AN, Moffatt TL, Wigle JT, Wigle TJ (2019). Exercise-induced increases in the expression and activity of cardiac sarcoplasmic reticulum calcium ATPase 2 is attenuated in AMPKα2 kinase-dead mice. Can J Physiol Pharmacol.

[23] Richter EA, Ruderman NB (2009). AMPK and the biochemistry of exercise: implications for human health and disease. Biochem J.

[24] Wadley G, Lee-Young RS, Canny BJ, Wasuntarawat C, Chen Z-P, Hargreaves M (2006). Effect of exercise intensity and hypoxia on skeletal muscle AMPK signaling and substrate metabolism in humans. Am J Physiol Endocrinol Metab.

[25] Imai S-I, Armstrong CM, Kaeberlein M, Guarente L (2000). Transcriptional silencing and longevity protein Sir2 is an NAD-dependent histone deacetylase. Nature.

[26] Custodero C, Saini SK, Shin MJ, Jeon YK, Christou DD, McDermott MM (2020). Nicotinamide riboside—A missing piece in the puzzle of exercise therapy for older adults?. Exp Gerontol.

[27] Kourtzidis IA, Stoupas AT, Gioris IS, Veskoukis AS, Margaritelis NV, Tsantarliotou M (2016). The NAD+ precursor nicotinamide riboside decreases exercise performance in rats. J Int Soc Sports Nutr.

[28] Wang X, Li B, Liu L, Zhang L, Ma T, Guo L (2021). Nicotinamide adenine dinucleotide treatment alleviates the symptoms of experimental autoimmune encephalomyelitis by activating autophagy and inhibiting the NLRP3 inflammasome. Int Immunopharmacol.

[29] Revollo JR, Grimm AA, Imai S-i (2004). The NAD biosynthesis pathway mediated by nicotinamide phosphoribosyltransferase regulates Sir2 activity in mammalian cells. J Biol Chem.

[30] Shibata K, Matsumoto K, Fushiki T, Sugimoto E (1994). Effects of exercise on the metabolism of NAD in rats. Biosci Biotechnol Biochem.

